# Clinical Outcomes of Trans-Sacral Epiduroscopic Laser Decompression (SELD) in Patients with Lumbar Disc Herniation

**DOI:** 10.1155/2020/1537875

**Published:** 2020-06-01

**Authors:** Seong Son, Sang Gu Lee, Yong Ahn, Woo Kyung Kim

**Affiliations:** Department of Neurosurgery, Gil Medical Center, Gachon University College of Medicine, Seongnam, Republic of Korea

## Abstract

**Objective:**

Nowadays, trans-sacral epiduroscopic laser decompression (SELD) using slender epiduroscopy and laser is one of the preferred options for minimally invasive treatment in lumbar disc diseases. However, SELD is still in the initial stages of the global field of spine surgery, and the clinical outcomes in patients with lumbar disc herniation are not established yet. Therefore, the authors investigated patients undergoing SELD to report the clinical results.

**Methods:**

Between November 2015 and November 2018, a total of 82 patients who underwent single-level SELD for lumbar disc herniation with a minimum follow-up of 6.0 months were enrolled. A retrospective review of clinical data was conducted. Clinical outcomes were evaluated using the visual analogue scale (VAS) for low back and leg pain and Odom's criteria. Also, surgical outcomes, including complications and symptom recurrences, and radiological outcomes were analyzed.

**Results:**

Low back pain and leg pain as determined by the VAS improved from an average of 5.43 ± 1.73 and 6.10 ± 1.67 to 2.80 ± 1.43 and 3.58 ± 2.08 at the final follow-up (*p* < 0.001). According to Odom's criteria, the success rate defined as excellent or good results at the final follow-up was 58.5%. There were no surgery-related complications such as neurologic deficits, infection, or epidural hematomas, except for transient mild paralysis in 3 patients and procedure-related nuchal pain in 2 patients. The rate of additional procedures was 17.0% (6 patients received revision surgery and 8 patients received an additional nerve block) during the follow-up.

**Conclusion:**

Our findings showed that SELD for lumbar herniated disc disease achieved less favorable clinical outcomes compared with those of previous studies. Further study is needed to clarify the influencing factors on the clinical outcomes of SELD.

## 1. Introduction

Epiduroscopy, also known as epidural spinal endoscopy, is defined as the percutaneous minimally invasive investigation of the epidural space with the assistance of a flexible endoscope through the sacral hiatus. It permits various clinical applications in the epidural space of the lumbosacral spine, such as epidural catheter placement and diagnosis, decompression of disc herniation, epidural adhesiolysis, delivery of epidural drug agents, and spinal cord stimulation electrode implants.

Epiduroscopy has been steadily and dramatically developed over the last century, achieving various milestones during the epiduroscopic surgery era. The first real endoscope was invented by Adolf Kussmaul in 1868, which led to the milestone development of a flexible fiberglass endoscope in 1958 by Hirschowitz [1]. Burman introduced the concept of epiduroscopy in 1931, and Stern first used clinical epiduroscopy for the diagnosis of various epidural lesions, including disc herniation, neuritis, and venous congestion [2]. Since the late 1960s, several pioneers, including Saberski, Ooi, Blomberg, Olsson, Holstrom, and Mollmann et al., developed the concept of spinal epiduroscopy through conducting human autopsies of the spine, and they reported on the clinical use of endoscopy via the sacral hiatus approach [3]. After that, in the 1990s, researchers, including Leu and Shutse et al., reported on the clinical usefulness and outcomes of epiduroscopy for the treatment of spinal disease [4, 5].

In 1996, the United States Food and Drug Administration eventually approved Myelotec Myeloscopy (Myelotec, Inc., Great Neck, NY), a modern epiduroscopic technique via the sacral hiatus, to visualize and treat spinal epidural pathology. After that, alongside small-caliber flexible optics and light sources, video-guided catheter and video systems, and laser technology, the present trans-sacral epiduroscopic decompression (SELD) technique was developed to treat the symptomatic pathology of the lumbosacral spine.

Since 2000s, in the early days of SELD, some reports have suggested its clinical application in various lumbar spinal diseases to not only disc herniation but also adhesion or fibrosis around nerve roots, failed back surgery, and even spinal stenosis [6–15]. Based on the principle of using lasers to condense hydrated disc herniation and the accumulated results of the previous literature, the indications of SELD were narrowed to soft disc herniations [16]. However, few reports have systematically reported the clinical results of SELD for lumbar disc herniation treatment.

In this paper, we reviewed the clinical, surgical, and radiological outcomes of SELD in patients with mild-to-moderate lumbar disc herniation with a follow-up of minimum 6 months.

## 2. Materials and Methods

### 2.1. Indications and Patient Population

The study was approved by the Institutional Review Board of our institute (GAIRB2018-214).

The indications for SELD were as follows: (1) magnetic resonance imaging (MRI) confirming mild-to-moderate soft disc herniation features concordant with the clinical symptoms and (2) persistent low back pain and/or radicular leg pain despite adequate conservative treatment or severe pain making daily life challenging. In addition, the contraindications for SELD were as follows: (1) cauda equina syndrome or severe paresis (motor grade 3 or less), (2) hard disc, (3) foraminal disc herniation which is inaccessible using SELD, (3) significant spinal stenosis or instability, (4) infection, (5) hemorrhagic diathesis, and (6) catheter-inaccessible anatomical abnormalities such as anomaly of sacral hiatus or peridural cyst.

**Figure 1:**
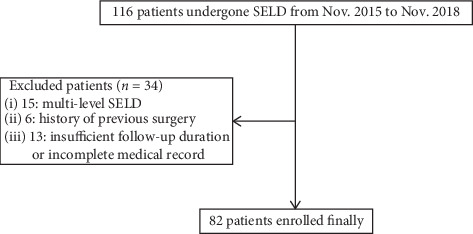
Flowsheet of patient selection.

From November 2015 to November 2018, among the 116 patients who underwent SELD in a single institution, 82 patients were retrospectively enrolled in the final cohort. The study exclusion criteria were as follows: (1) multilevel procedure, (2) previous history of lumbar surgery, and (3) insufficient follow-up duration of 6 months or an incomplete medical record (Figure 1).

Data were based on regular follow-up data obtained from the medical records, and patients who did not visit regularly or whose follow-up period was less than 6 months were contacted by phone to participate in clinical surveys and X-rays.

### 2.2. Operative Technique

The procedure was performed in the awakened state under local anesthesia using lidocaine with epinephrine over the sacral hiatus with monitored vital signs. Each patient was placed in the prone position on a radiolucent table with a Wilson's frame to decrease lumbosacral lordosis and intra-abdominal pressure.

A 5 mm-sized tiny skin incision was made on the sacral hiatus, followed by a sacrococcygeal ligament puncture using a trocar under fluoroscopic guidance. After the advancement of the trocar to the S2-3 level, a video-guided catheter of 3.2 mm in diameter (Spinaut V, Imedicom Inc., Seoul, Korea) containing two lumens (1.2 mm in diameter) was inserted through the trocar to the target level. Consequently, the video-guided catheter was advanced to the target level using bidirectional steering characteristics and the injection of radio-opaque dye via an infusion port, and fluoroscopic pictures were obtained to verify the position of the catheter in the ventral epidural space and confirm the outline of the herniated disc and flow obstruction caused by disc herniation and adhesion around the pathologic site. Through the video-guided catheter, the flexible epiduroscope of 1.0 mm in diameter (Spinaut S, Imedicom Inc., Seoul, Korea) and the flexible fiber of a Ho:YAG laser of 550 *μ*m in diameter were advanced into the end of the catheter to visualize the epidural space and condense pathologic lesion.

The Ho:YAG laser generated by Litho (Quanta system Inc, Milan, Italy) was used in this study because of its high-quality ablation of the hydrated disc without significant thermal injury to the neural structures [17]. The laser has a 2100 nm wavelength and a 0.4 mm depth of penetration [6].

Under direct vision via a flexible epiduroscope, after confirmation that the tip of the catheter was located at the most inferior part of the herniated disc covered by the posterior longitudinal ligament, adhesiolysis around the target site and laser ablation of the herniated disc were performed. First, the bulging posterior longitudinal ligament was shrunk using a Ho:YAG laser at 2.5 W (0.5 J and 5 Hz), checking the patient's response. Once the patient tolerated the low-grade laser, the posterior longitudinal ligament was penetrated using the laser of 5 W (0.5 J and 10 Hz). Then, the herniated disc under the posterior longitudinal ligament was shrunk and decompressed by the high-energy laser of 8–10 W (0.8–1.0 J and 10 Hz). Protruded or ruptured discs were decompressed until the direct images confirmed a decompressed nerve root or thecal sac.

As the herniated disc decreased, the epidural space between the dura and pathologic lesion became wider. Repeated epidurograms should show a flattened outline and free flow at the target site after sufficient decompression.

The wounds were closed by one-point subcutaneous suture and skin tape.

All procedures were performed by one surgeon.

### 2.3. Outcome Evaluation

Demographic data and baseline characteristics, including age, sex, occupation, smoking status, alcohol consumption, body mass index, past medical history, previous history of epidural block, trauma history, surgical level, and preoperative symptom duration, were evaluated. Lumbar magnetic resonance imaging was performed prior to surgery, and preoperative state including surgery level, grade of disc degeneration using the Pfirrmann grade [18], disc height, presence of annular tearing known as high-intensity zone, degree of disc protrusion (bulging, protrusion, or extrusion), the volume of disc herniation (determined as transverse diameter × depth × height of disc herniation × 1/2), location of protruded disc (central, right, or left), degree of combined stenosis (none/mild/moderate/severe), and degree of root compression (abutting/displace/near obliteration/obliteration) were evaluated.

Clinical outcomes were assessed using the visual analogue scale (VAS) scores of low back pain and leg pain. Data were collected preoperatively and at each follow-up visit (at 1 week, 1 month, and 6 months). Patient satisfaction was surveyed using Odom's criteria at each follow-up visit (at 1 week, 1 month, and 6 months).

Surgical outcomes were evaluated using operation time; surgical failures; surgical complications, such as durotomy, neurologic deterioration, and surgical site infections; hospital length of stay; and return-to-work timing. Long-term surgical outcomes were assessed according to performance of additional procedures, including revision surgeries, nerve root blocks, and epidural blocks during the follow-up period.

Plain radiography, including dynamic radiography, was performed at preoperation and 6 months after surgery to assess the change in lumbar alignment. Segmental angle and range of motion at surgery level, and total lumbar lordosis (measured using Cobb's method) were used to evaluate radiological outcomes.

### 2.4. Statistical Analysis

Data management and statistical analysis were performed using SPSS version 23.0 (SPSS Inc., Chicago, IL, USA). Pearson's chi square test, Wilcoxon's signed-rank test, the paired samples *t*-test, Friedman's test (a nonparametric multiple comparison test), one-way analysis of variance (ANOVA), independent *t*-test, and nonparametric Mann–Whitney *U*-test were used for univariate comparison according to characteristics of the factors. Kaplan–Meier survival analysis was used for the analysis of survival without additional procedures.

Results were expressed as means ± standard deviations, medians with ranges, or mean and 95% confidence interval (CI), and statistical significance was accepted for *p* values of <0.05.

## 3. Results

### 3.1. Demographic Data and Baseline Characteristics

The 82 study subjects comprised 52 men and 30 women, with an overall mean age of 40.78 ± 15.24 years. The mean body mass index was 24.15 ± 3.99, and 6 patients (7.3%) had diabetes mellitus.

The median duration of symptoms was 1.0 week (range, 0.1–12.0). Twelve patients (14.6%) had a minor trauma history related to symptom aggravation, and 26 patients (31.7%) had a low back pain dominant symptom other than radicular leg pain. The median follow-up period was 23.0 months (range, 6.0–30.0) (Table 1).

According to preoperative MRI, the surgical levels were the following: L3-4 in 6 patients, L4-5 in 22 patients, and L5–S1 in 54 patients. A high-intensity zone was revealed in 28 patients (34.2%), and the herniated disc volume was 0.30 ± 0.12 mL (Table 2).

### 3.2. Clinical Outcomes

**Figure 2:**
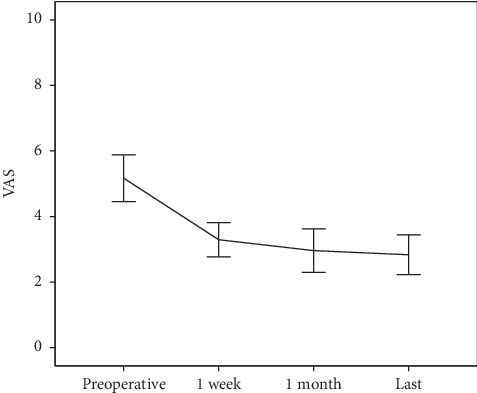
Visual analogue scale (VAS) for low back pain.

For all 82 study subjects, the mean preoperative VAS for low back pain was 5.43 ± 1.73, and this decreased to 3.22 ± 1.44 at 1-week postoperation, 2.59 ± 1.56 at 1-month postoperation, and 2.8 ± 1.43 at the final follow-up (*p* < 0.001, one-way ANOVA). Preoperation to 1-week postoperation, preoperation to 1-month postoperation, and preoperation to the final follow-up differences in VAS for low back pain were significant (2.22 ± 0.34 [95% CI, 1.32–3.12], 2.85 ± 0.36 [95% CI, 1.91–3.79], and 2.61 ± 0.40 [95% CI, 1.56–3.65], *p* < 0.001, respectively, ANOVA with post hoc), but the differences between 1-week postoperation, 1-month postoperation, and the final follow-up of VAS for low back pain were not significant (Table 3 and Figure 2).

**Figure 3:**
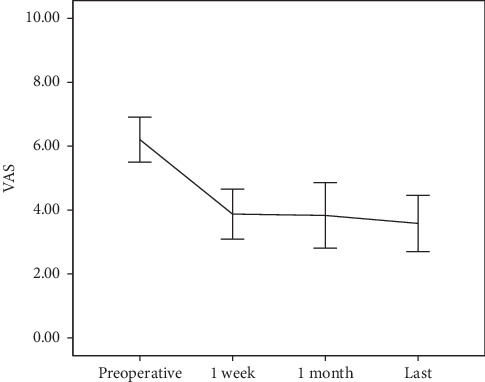
Visual analogue scale (VAS) for radicular leg pain.

The mean preoperative VAS for leg pain was 6.10 ± 1.67, and this decreased to 3.90 ± 1.83 at 1-week postoperation, 3.35 ± 2.36 1-month postoperation, and 3.58 ± 2.08 at the final follow-up (*p* < 0.001, one-way ANOVA). Preoperation to 1-week postoperation, preoperation to 1-month postoperation, and preoperation to the final follow-up differences in VAS for leg pain were significant (2.20 ± 0.44 [95% CI 1.06–3.33], 2.74 ± 0.46 [95% CI 1.55–3.93], and 2.51 ± 0.51 [95% CI 1.20–3.83], *p* < 0.001, respectively, ANOVA with post hoc), but the differences between 1-week postoperation, 1-month postoperation and final follow-up of VAS for leg pain were not significant (Table 3 and Figure 3).

According to Odom's criteria, the results were excellent in 10 (12.2%) and good in 40 patients (48.8%) at 1-week after the operation, excellent in 20 (24.4%) and good in 28 (34.1%) at 1-month after the operation, and excellent in 16 (19.5%) and good in 32 (39.0%) at the final follow-up. In other words, the success rate of the surgery (excellent or good according to Odom's criteria) was 61.0% at 1-week after operation, 58.5% at 1-month after operation, and 58.5% at the final follow-up. Odom's criteria distributions at all points of time after surgery were not significantly different (*p*=0.551, Pearson's chi-square test) (Table 3).

### 3.3. Surgical Outcomes

The median operation time was 50.0 minutes (range, 30.0–100.0), the mean hospital stay was 3.60 ± 0.80 days, and the mean time return-to-work was 15.41 ± 6.92 days (Table 4).

Surgical complications occurred in 7 patients (8.5%), including 4 patients with transient headache or nuchal pain during the procedure, 2 patients with transient motor weakness, and 1 patient with dura puncture during the procedure. Fortunately, there were no surgical site infections or permanent neurologic deficits after the procedure. In addition, there was no perioperative morbidity related to the procedure, such as a cardiopulmonary problem or deep vein thrombosis.

Eight patients (9.8%) underwent an additional epidural block or root block for persistent pain or pain recurrence during the follow-up, and the pain was appreciably relieved in all of these patients. Six patients (7.3%) underwent revision surgery at the index level due to aggravation of symptoms during their follow-ups (discectomy for 5 patients and fusion for 1 patient) (Table 4).

**Figure 4:**
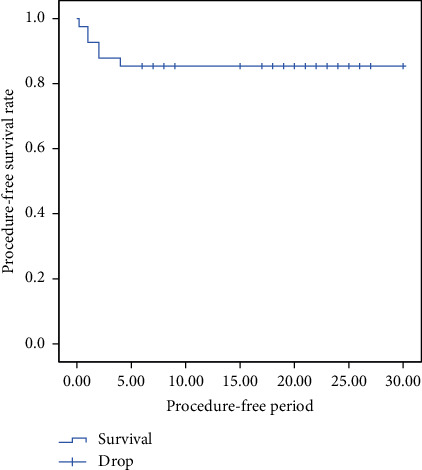
Kaplan–Meier survival analysis of survival without additional procedures.

The 6-month procedure-free survival rate was 82.9%, and the mean overall time before an additional procedure, including a nerve block or revision surgery, was 25.86 ± 1.56 months (95% CI, 22.79–28.92) (Figure 4).

### 3.4. Radiological Outcomes

All radiological findings, including disc height of index level, segmental angle of index level, range of motion of index level, and total lumbar lordosis, were not significantly different between the preoperation and the final follow-up (Table 5).

## 4. Discussion

After the United States Food and Drug Administration approval of epiduroscopy, the concept of SELD was developed, and the literatures reported the clinical outcomes of SELD in not only disc herniation but also in spinal stenosis, adhesion, chronic low back pain, failed back syndrome, and cystic lesions in the lumbosacral spine [7–15, 19, 20]. However, considering the principles of SELD, the distinctive difference from conventional procedures of drug injection or adhesiolysis like epidural neuroplasty is the effect of laser ablation on hydrated soft tissue [17–21]. In other words, whereas the function of drug delivery or adhesiolysis could create a transient effect as in other procedures, laser ablation of soft disc herniation could lead to the permanent effect of decompression [22–24]. According to this concept, the optimal indication of SELD may be considered to be soft disc herniation rather than stenosis, adhesion, or failed back syndrome [16].

However, there were few reports about the clinical outcomes of SELD for disc herniation, and even if these existed, the follow-up duration was often short or there was a flaw in the reliability of the results. Several previous reports stated that the clinical outcomes of SELD were favorable even compared with those of open discectomy, as there was a significant decrease of low back pain or radiating leg pain, patient satisfaction higher than 70%, low rates of incomplete decompression, and a low recurrence rate [16, 17, 22–25].

However, according to this study, the clinical results of the patients were not all favorable. Although the mean VAS of low back pain and leg pain decreased significantly, the satisfaction and surgical failure rates were not all good. According to Odom's criteria, the patient satisfaction rate was 58.5%, and surgical failure rate, including additional nerve blocks or revision surgeries, was 17.1% during the follow-up period of a minimum of 6 months. This result was not favorable compared with not only the previous study of SELD but also the results of other surgical technique for lumbar disc herniation [16, 17, 22–25].

The authors raise several hypotheses about the reasons for this discordance with previous studies. First, the effect of decompression after laser ablation might be lower than expected. Although the surgeon confirmed decompression of the lesion during the procedure, the objective reduction of the herniated disc could be minimal in immediate postoperative MRI [23]. Also, because of the delayed effects of laser ablation and dehydration of herniated soft disc, clinical results could vary in many patients [16]. However, the final cohort in this study had a minimum of 6 months of follow-up, and this sufficient follow-up period could rule out this hypothesis.

Second, the learning curve of SELD of surgeons could affect the result. The result may be less favorable in the early stages of clinical application than in the later stages. Further study is needed to clarify the effect of surgeon's learning curve in clinical outcomes.

Third, the selection of different patients could cause varied clinical outcomes. Even in the similar patients with single-level soft disc herniation, differences in detailed baseline characteristics such as demographic data, including age and past medical history, disc level, location of pathology, morphology of pathology, or disc degeneration could affect the clinical outcome. To clarify whether these baseline factors have a positive or negative effect on outcomes, further research is needed.

This study has some limitations. Due to its retrospective nature, it was impossible to control all variations. Nevertheless, we tried to minimize errors by precluding the variables associated with results; for example, we excluded patients with multilevel procedures or previous histories of lumbar spine surgery and who received insufficient follow-up. Also, the number of patients in the final cohort was not large enough, and the study was conducted at a single center. However, this single center study could maintain the quality of follow-up and exclude the factor of the diversity of surgeons. Of course, further studies with large numbers of subjects are mandatory to confirm the clinical results and to clarify the influencing factors on the clinical outcomes of SELD.

## 5. Conclusion

The clinical outcomes of SELD with a minimum 6 month follow-up were not favorable compared to those of previous studies, as patient satisfaction was 58.5% and additional procedure rate was 17.1%. According to these results, we believe that there could be several reasons for this variation in clinical outcomes, including a learning curve and baseline influencing factors on outcomes; thus further study with a larger cohort is needed.

## Figures and Tables

**Table 1:** Demographic data and baseline characteristics.

Characteristics	Number (*n* = 82)
Age	40.78 ± 15.24 (95% CI 35.97–45.59)
Sex (male/female)	52/30 (63.41%)
Occupation (white/blue/etc.)	36/18/28
Smoking (yes/no)	26/56 (31.7%)
Pack-years	3.74 (95% CI 1.50–5.99)
Alcohol consumption (g/week)	0 (range, 0–120.0)
Height (cm)	169.46 ± 10.24 (95% CI 166.23–172.70)
Weight (kg)	69.50 ± 13.77 (95% CI 65.15–73.84)
Body mass index (kg/m^2^)	24.15 ± 3.99 (95% CI 22.88–25.40)
Diabetes mellitus (yes/no)	6/76 (7.32%)
Hypertension (yes/no)	18/64 (21.95%)
Symptom duration (weeks)	1.0 (range, 0.1–12.0)
Admission route (outpatient/emergency room)	72/10
Previous block (yes/no)	48/34 (58.54%)
Trauma history (yes/no)	12/70 (14.63%)
Dominant symptom (low back pain/leg pain)	26/56
Follow-up duration (months)	23.0 (range, 6.0–30.0)

**Table 2:** Baseline characteristics determined by preoperative magnetic resonance imaging and intraoperative findings.

Characteristics	Number (*n* = 82)
Surgical level (L3-4/L4-5/L5–S1)	6/22/54
Pfirrmann grade (I/II/III/IV)	0/22/50/10
High-intensity zone (yes/no)	28/54 (34.2%)
Disc morphology (bulging/protrusion/extrusion)	10/46/26
Location of herniation (central/right/left)	26/20/36
Degree of canal compromise (mild/moderate/severe)	60/22/0
Root compression grade (abutting/displace/near obliteration/obliteration)	42/28/10/2
Herniated disc volume (mL)	0.30 ± 0.12 (95% CI 0.26–0.34)
Degree of stenosis (none/mild/moderate/severe)	54/26/2/0
Adhesion during surgery (mild/moderate/severe)	5/22/45

**Table 3:** Clinical outcomes.

Characteristics		*p* value
VAS for low back pain		<0.001^†^
Preoperative	5.43 ± 1.73	
1 week	3.22 ± 1.44	
1 month	2.59 ± 1.56	
Final follow-up	2.80 ± 1.43	
ΔVAS for low back pain		
Preoperative to 1 week	2.22 ± 0.34 (95% CI, 1.32–3.12)	<0.001^†^
Preoperative to 1 month	2.85 ± 0.36 (95% CI, 1.91–3.79)	<0.001^†^
Preoperative to final *f*/*u*	2.61 ± 0.40 (95% CI, 1.56–3.65)	<0.001^†^
1 week to 1 month	0.63 ± 0.36 (95% CI, −0.31–1.57)	0.304^†^
1 week to final follow-up	0.39 ± 0.40 (95% CI, −0.66–1.43)	0.770^†^
1 month to final follow-up	−0.25 ± 0.42 (95% CI, −1.33–0.84)	0.935^†^

VAS for leg pain		<0.001^†^
Preop	6.10 ± 1.67	
1 week	3.90 ± 1.83	
1 month	3.35 ± 2.36	
Final follow-up	3.58 ± 2.08	

ΔVAS for leg pain		
Preop to 1 week	2.20 ± 0.44 (95% CI, 1.06–3.33)	<0.001^†^
Preop to 1 month	2.74 ± 0.46 (95% CI, 1.55–3.93)	<0.001^†^
Preop to final *f*/*u*	2.51 ± 0.51 (95% CI, 1.20–3.83)	<0.001^†^
1 week to 1 month	0.55 ± 0.46 (95% CI, −0.64–1.74)	0.627^†^
1 week to final *f*/*u*	0.32 ± 0.51 (95% CI, −1.00–1.64)	0.922^†^
1 month to final *f*/*u*	−0.23 ± 0.53 (95% CI, −1.60–1.14)	0.972^†^

Odom's criteria		0.551^‡^
1 week, excellent/good/fair/poor	10/40/30/2	
1 month, excellent/good/fair/poor	20/28/34/0	
Final follow-up, excellent/good/fair/poor	16/32/30/4	
Success rate at 1 week	61.0% (50 patients)	
Success rate at 1 month	58.5% (48 patients)	
Success rate at final follow-up	58.5% (48 patients)	

^†^One-way ANOVA. ^‡^Pearson's chi-square test.

**Table 4:** Surgical outcomes.

Characteristics	Number (*n* = 82)
Operation time (minutes)	50.0 (range, 30.0–100.0)
Hospital stay (days)	3.60 ± 0.80
Time to return-to-work (days)	15.41 ± 6.92

Surgical complication	7 (8.5%)
Headache or nuchal pain during procedure	4 (4.9%)
Transient motor weakness	2 (2.4%)
Dural puncture	1 (1.2%)
Additional procedure	14 (17.1%)
Additional epidural block	8 (9.8%)
Revision surgery	6 (7.3%)

**Table 5:** Radiological outcomes.

Characteristics		*p* value
Disc height (mm)		
Preoperative	18.21 ± 1.18	
6 months	18.02 ± 1.44	
Δ preoperative − 6 months	0.21 ± 1.25 (95% CI −1.43–1.96)	0.670^†^

Segmental angle at the surgery level (°)		
Preoperative	7.70 ± 4.69	
6 months	7.97 ± 4.20	
Δ preoperative − 6 months	−0.28 ± 2.23 (95% CI, −1.26–0.72)	0.571^†^

Range of motion at the surgery level (°)		
Preoperative	5.94 ± 4.48	
6 months	7.32 ± 6.16	
Δ preoperative − 6 months	−1.38 ± 6.23 (95% CI, −4.14–1.39)	0.312^†^

Total lumbar lordosis (°)		
Preoperative	31.25 ± 16.44	
6 months	35.83 ± 11.01	
Δ preoperative − 6 months	−1.38 ± 10.73 (95% CI, −9.33–0.18)	0.058^†^

^†^Paired *t*-test.

## Data Availability

The data used to support the findings of this study are included within the article.
